# Generating synthetic contrast enhancement from non-contrast chest computed tomography using a generative adversarial network

**DOI:** 10.1038/s41598-021-00058-3

**Published:** 2021-10-14

**Authors:** Jae Won Choi, Yeon Jin Cho, Ji Young Ha, Seul Bi Lee, Seunghyun Lee, Young Hun Choi, Jung-Eun Cheon, Woo Sun Kim

**Affiliations:** 1 Department of Radiology, Seoul National University Hospital, 101 Daehak-ro, Jongno-gu, Seoul, 03080 Korea; 2 Department of Radiology, Seoul National University College of Medicine, 103 Daehak-ro, Jongno-gu, Seoul, 03080 Korea; 3 Department of Radiology, Gyeongsang National University Changwon Hospital, Changwon, 51472 Korea; 4 Institute of Radiation Medicine, Seoul National University Medical Research Center, 103 Daehak-ro, Jongno-gu, Seoul, 03080 Korea

**Keywords:** Medical imaging, Tomography

## Abstract

This study aimed to evaluate a deep learning model for generating synthetic contrast-enhanced CT (sCECT) from non-contrast chest CT (NCCT). A deep learning model was applied to generate sCECT from NCCT. We collected three separate data sets, the development set (n = 25) for model training and tuning, test set 1 (n = 25) for technical evaluation, and test set 2 (n = 12) for clinical utility evaluation. In test set 1, image similarity metrics were calculated. In test set 2, the lesion contrast-to-noise ratio of the mediastinal lymph nodes was measured, and an observer study was conducted to compare lesion conspicuity. Comparisons were performed using the paired t-test or Wilcoxon signed-rank test. In test set 1, sCECT showed a lower mean absolute error (41.72 vs 48.74; *P* < .001), higher peak signal-to-noise ratio (17.44 vs 15.97; *P* < .001), higher multiscale structural similarity index measurement (0.84 vs 0.81; *P* < .001), and lower learned perceptual image patch similarity metric (0.14 vs 0.15; *P* < .001) than NCCT. In test set 2, the contrast-to-noise ratio of the mediastinal lymph nodes was higher in the sCECT group than in the NCCT group (6.15 ± 5.18 vs 0.74 ± 0.69; *P* < .001). The observer study showed for all reviewers higher lesion conspicuity in NCCT with sCECT than in NCCT alone (*P* ≤ .001). Synthetic CECT generated from NCCT improves the depiction of mediastinal lymph nodes.

## Introduction

Iodinated contrast media are widely used in computed tomography (CT) to enhance tissue contrast, making it easier to evaluate anatomic structures and pathologies. However, iodinated contrast media have potential adverse effects varying from minor physiologic reactions to severe life-threatening situations, although their incidence has decreased with the development of low-osmolar and non-ionic contrast agents^1,2^. Many chest CT examinations, which are undeniably crucial diagnostic tools to evaluate thoracic disorders, are non-contrast CT (NCCT), especially for screening purposes or initial evaluation. The use of contrast in chest CT is often unnecessary for detecting lung parenchymal lesions. However, contrast-enhanced CT (CECT) plays a critical role in the detailed assessment of the mediastinum, pleura, and vessels.

In recent years, deep learning has been applied to various tasks in medical imaging, including automatic lesion detection, segmentation, or image quality improvement. One of the most interesting current implementations of deep learning in medical imaging is synthetic image generation and the generative adversarial network (GAN) is considered state-of-the-art for such a task^3,4^. A recent study used a deep learning algorithm to synthesize contrast enhancement from non-contrast cardiac CT^5^. However, the authors only used slices where the heart was present and mainly focused on delineating the left cardiac chamber. We think that generating synthetic contrast enhancement from a full-volume NCCT without additional scan or intravenous contrast injection would prove more useful in clinical practice without any added risks to the patients.

This study aimed to propose and evaluate a deep learning approach using GAN for generating synthetic contrast-enhanced CT (sCECT) images from non-contrast chest CT.

## Methods

This retrospective study was approved by the Seoul National University Hospital Institutional Review Board (SNUH, IRB no. 1910-152-1073) and the Institutional Review Board of Gyeongsang National University Changwon Hospital (GNUCH, IRB no. 2020-07-011). Both institutional review boards waived the requirement of informed consent for the study. All methods were performed in accordance with the relevant guidelines and regulations.

### Data acquisition

**Figure 1:**
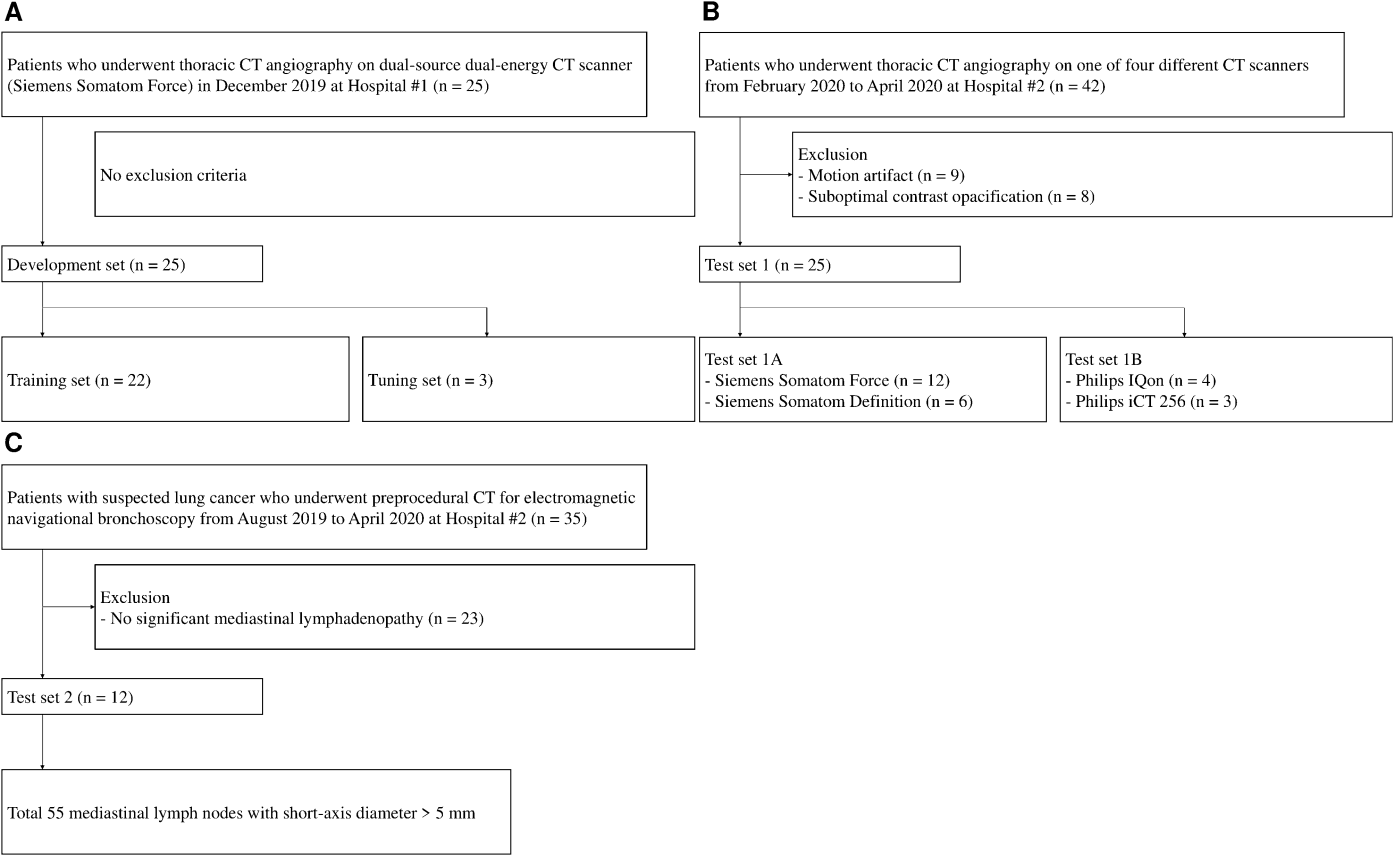
Flowchart depicting patient inclusion and exclusion. Development set (**A**), test set 1 (**B**), and test set 2 (**C**).

We collected three separate data sets, the development set (for model training and tuning) from Hospital #1 (GNUCH) and test sets 1 (for technical evaluation) and 2 (for clinical utility evaluation) from Hospital #2 (SNUH). Patient inclusion is shown in Fig. 1. The development set included consecutive patients who underwent dual-energy thoracic CT angiography (Somatom Force; Siemens, Erlangen, Germany) in December 2019. There were no exclusion criteria in the development set. The development set was randomly split (9:1 ratio) into training and tuning sets. Test set 1 included consecutive patients who underwent thoracic CT angiography on various CT scanners between February 2020 and April 2020. Patients with CT examinations with motion artifacts or suboptimal contrast opacification were excluded. Test set 1 was divided into test sets 1A and 1B based on the CT vendor. Patients whose CT vendor was the same as that in the development set (Siemens, Erlangen, Germany) were included in test set 1A, and test set 1B consisted of the remaining patients.

For evaluation of clinical utility, a separate test set with clinical relevance had to be constructed. Test set 2 comprised consecutive patients with suspected lung cancer who underwent preprocedural CT examinations for electromagnetic navigational bronchoscopy between August 2019 and April 2020. Among them, patients with at least one mediastinal lymph node with a short-axis diameter > 1 cm (significant lymphadenopathy) were included. At Hospital #2, patients underwent pre-bronchoscopic CT examinations consisting of a pre-contrast and a routine contrast-enhanced scan on a designated single CT scanner (IQon; Philips, Andover, Massachusetts). We obtained paired virtual non-contrast (VNC) and CECT data for the development set and paired NCCT and CECT data for the test sets.

### Image preprocessing

All axial CT images were downloaded in Digital Imaging and Communications in Medicine (DICOM) format from picture archiving and communication systems (PACS) after anonymization. The size of all CT images was the same (512 × 512 pixels), and we did not resize the images. The original CT images ranged from − 1024 to over 3000 HU. We acquired three greyscale images from each axial CT image by applying three different window settings, normalized them to a range of − 1 to 1, and combined them into a 3-channel image. We tried various combinations of CT window settings in a preliminary study, and eventually, those we used in this study were as follows: lung/bone window (window width, 2000 HU; level, 0 HU), vascular window (window width, 1000 HU; level, 200 HU), and mediastinal window (window width, 500 HU; level, 50 HU).

### Lesion annotation

A board-certified radiologist (Y.H.C.) and a radiology resident (J.W.C.) with 16 and 4 years of experience, respectively, reviewed the CECT images of test set 2 and annotated mediastinal lymph nodes with a short-axis diameter > 5 mm. The measurement of lesion contrast-to-noise ratio (CNR) and the observer study were based on these annotations. There were a total of 55 annotated mediastinal lymph nodes (mean short-axis diameter, 8.62 ± 2.47 mm).

### Deep learning model development

Source code for training and inference of our deep learning model is available at https://github.com/jwc-rad/pix2pix3D-CT. The architecture of our deep learning model is illustrated in Supplementary Fig. S1. The basic structure of our proposed model is identical to that of the original pix2pix model^6^, except that the 2D convolutional layers are replaced with their 3D counterparts. The proposed model consists of a generator network and a discriminator network, as in a conventional generative adversarial network (GAN)^4^. The generator network is an encoder-decoder convolutional neural network with skip connections (U-Net)^7^, with an input and output size of 512 × 512 × 16. Each encoder block is composed of a convolution with stride, Leaky ReLu ^8^, and instance normalization^9^, whereas each decoder block consists of an upsampling layer, convolution with stride, ReLu^8^, instance normalization, and a skip connection. To reduce the checkerboard artifacts of a GAN, the model uses resize-convolution with nearest-neighbor interpolation for the upsampling layer of the decoder block^10^. A skip connection concatenates encoder block *i* and decoder block *n-i*, where *n* is the total number of blocks, and passes the output to a ReLu activation layer. The final output layer of the generator network uses the Tanh function^8^. The discriminator network is a PatchGAN^6^ that classifies each 70 × 70 × 4 pixel patch as real or fake and whose convolutional module is identical to the encoder block of the generator network.

### Training the deep learning model

For the deep learning implementation, we used Keras (version 2.1.5, https://keras.io/), a Python-based high-level deep-learning library, run on top of Tensorflow (version 1.4.0; Google Brain Team, Mountain View, CA). For the model training, we followed a standard adversarial approach of alternating training steps on the discriminator network and generator network^4^. The objective function of the proposed model is a weighted sum of the GAN loss and L1 loss of the generator network. Although the ratio of weights is a hyperparameter that may be altered for optimization, we set it to a fixed ratio of 1:100, as in the original pix2pix paper^6^. To stabilize the training process, we adopted general techniques for training GANs, proposed by Ian Goodfellow^11^ and Radford et al.^8^, including one-sided label smoothing and the use of the Adam optimizer^12^. For data augmentation, we performed a random vertical and horizontal shift of up to 50 pixels on the input before feeding the image into the model. The training parameters were as follows: Adam optimizer with a learning rate of 0.0002, beta 1 of 0.5, and a decay rate of 0.1 after the first 10 epochs; a batch size of 1; and a total of 20 epochs. Each epoch covered all possible sets of 16 consecutive axial images of the training set, which was approximately 3000 iterations. The entire training process took about 4 days on a cloud-based workstation with an NVIDIA Tesla V100 GPU (NVIDIA, Santa Clara, CA) and 26 GB RAM. As there is no gold standard objective measure for the performance of a GAN, we relied on the visual inspection of images synthesized by the generator network^6,13^. During the training phase, a radiologist (J.W.C.) monitored random samples of the generated images, and after the training process, he validated the results generated from the tuning set.

### Applying the deep learning model

For inference, we used only the generator network of our proposed model. The inference process was performed in the same manner for all data sets, regardless of whether the input was VNC or NCCT. As the size of the input was the same as in the training process, the direct synthesis of an entire CT volume of a patient was not possible. Instead, one slice at a time from the top, we repeated the application of the generator network to the next 16 consecutive slices while indexing the slice numbers. For a CT volume with a shape of 512 × 512 × N, running the generator network through the entire CT volume would first yield *N-15* arrays with a shape of 512 × 512 × 16 that partially overlap with one another. Then, the overlapping slices, which would be the images with the same indexed slice number, were averaged. Finally, we reconverted the first channel image (lung/bone window) of the averaged output to a synthetic CT image with a range of -1000 to 1000 HU.

### Image analysis: technical evaluation

We employed the mean absolute error (MAE), peak signal-to-noise ratio (PSNR)^14,15^, multiscale structural similarity index measurement (MS-SSIM)^16^, and learned perceptual image patch similarity metric (LPIPS)^17^ to perform a quantitative evaluation of the tuning set and test set 1. A lower MAE, higher PSNR, higher MS-SSIM, and lower LPIPS indicate higher similarity to the ground truth. MAE and PSNR reflect the absolute numerical difference between two images, whereas MS-SSIM correlates with similarity in the structural composition of pixels^14,18^. LPIPS is a more recently suggested metric of perceptual distance based on widely used pretrained deep neural networks^17,19^. For comparison, we calculated the metrics for both sCECT and input images (NCCT or VNC) in the mediastinal window (window width, 350 HU; level, 50 HU), each relative to the corresponding CECT images. We only included axial slices between the top of the aortic arch and the diaphragm for image similarity analysis.

### Image analysis: performance in depicting mediastinal lymph nodes

To explore the clinical utility of sCECT images, we evaluated the performance of sCECT in depicting mediastinal lymph nodes using test set 2. As a quantitative analysis, we measured the lesion CNR of the mediastinal lymph nodes. For each lesion, the measurement was performed on the axial slice of the contrast-enhanced CT, where the short-axis diameter was measured. We first drew a circular region of interest (ROI) inside the lesion, measuring 90% of the lesion’s short-axis diameter. Circular ROIs of the same size were additionally drawn inside the descending thoracic aorta and subcutaneous fat of the bilateral chest wall. The ROIs were then copied to the same locations on the non-contrast and synthetic contrast-enhanced axial images. The contrast-to-noise ratio (CNR) of all lesions was calculated as follows:

$$ {\text{Background }}\,{\text{noise}} = \sqrt {\frac{{{\text{SD}}_{{{\text{right}}\,{\text{fat}}}}^{2} + {\text{SD}}_{{{\text{left }}\,{\text{fat}}}}^{2} }}{2}} $$

$$ {\text{CNR}}_{{{\text{lesion}}}} = \frac{{\left| {{\text{HU}}_{{{\text{DTA}}}} - {\text{HU}}_{{{\text{lesion}}}} } \right|}}{{{\text{Background}}\,{\text{noise}}}} $$

where *HU* is the mean HU value of the ROI, *SD* is its standard deviation, and *DTA* is descending thoracic aorta.

For the qualitative analysis, two blinded board-certified radiologists (Y.J.C. and S.B.L. with 8 and 3 years of experience, respectively) participated in a three-session review of CT images with two-week intervals using a Digital Imaging and Communications in Medicine viewer (RadiAnt, version 2020.1; Medixant, Poznan, Poland). Each session consisted of NCCT, NCCT with sCECT, and CECT images, respectively, from each patient in test set 2 presented in random order. The reviewers were instructed to label mediastinal lymph nodes with a short-axis diameter > 5 mm and report lesion conspicuity on a 4-point scale (1, barely perceptible with presence debatable; 2, subtle finding but likely a lesion; 3, definite lesion detected; and 4, strikingly evident and easily detected)^20^. The conspicuity of undetected lesions was recorded as 0.

### Statistical analysis

For comparison of image similarity metrics and lesion CNR, we applied the paired t-test or the Wilcoxon signed-rank test according to the Shapiro–Wilk normality test. For the observer study, the detection rate of the lymph nodes was compared using the McNemar test and the differences in lesion conspicuity were evaluated using the Wilcoxon signed-rank test. Also, we evaluated lesion localization using the figures of merit (FOM) from jackknife alternative free-response receiver operating characteristic (JAFROC) analysis^21^. We report the results from the random reader, fixed case JAFROC analysis because of the small number of cases of our study. *P* < 0.05 was considered indicative of a statistically significant difference. All data were analyzed using MedCalc (version 12.7, MedCalc Software, Ostend, Belgium), scikit-learn library (version 0.20.3, https://scikit-learn.org/), and JAFROC software for Windows (version 4.2.1, WindowsJafroc, https://www.devchakraborty.com).

### Informed consent

This retrospective study was approved by the institutional review boards, which waived the need for patient informed consent.

## Results

### Patient characteristics

Patient characteristics and CT acquisition parameters are summarized in Table 1. A total of 62 patients (35 men, 27 women) with a median age of 67.5 years (interquartile range [IQR], 58–74 years) were enrolled. The development set comprised 25 patients (median age, 66 years [IQR, 53–79]; 13 men, 12 women). For the test set 1, among 42 patients who underwent thoracic CT angiography on one of four different CT scanners (Somatom Force and Somatom Definition, Siemens, Erlangen, Germany; IQon and iCT 256, Philips, Andover, Massachusetts) at Hospital #2, 17 patients were excluded due to motion artifacts (n = 9) and suboptimal contrast opacification (n = 8). The test set 1 included 25 patients (median age, 66 years [IQR, 58.5–72]; 14 men, 11 women). Among them, 18 patients, whose CT vendor was the same as that in the development set, were included in the test set 1A, and test set 1B consisted of the seven remaining patients. For test set 2, among 35 patients who underwent pre-bronchoscopic CT at Hospital #2, 23 patients without significant mediastinal lymphadenopathy were excluded. Thus, test set 2 comprised 12 patients (median age, 70.5 years [IQR, 67–76]; 8 men, 4 women) with a total of 55 mediastinal lymph nodes (mean short-axis diameter, 8.62 ± 2.47 mm).

**Table 1:** Patient characteristics and CT acquisition parameters.

	Development set (n = 25)	Test set 1A (n = 18)	Test set 1B (n = 7)	Test set 2 (n = 12)
**Patient characteristics**				
Age (y)*	66 (53–79)	65.5 (52–71)	68 (62–72)	70.5 (67–76)
Sex (male/female)	13/12	11/7	3/4	8/4
Clinical indication	Not specified	Not specified	Not specified	Suspected lung cancer
**CT scanning parameters**				
CT vendor	Siemens	Siemens	Philips	Philips
CT scanner	Somatom Force	Somatom Force, Somatom Definition	IQon, iCT 256	IQon
Tube voltage (kVp)	70/150 with tin filter	90–110	100–120	120
Collimation (mm)	96 × 0.6	32 or 96 × 0.6	64 or 128 × 0.625	64 × 0.625
Matrix	512 × 512	512 × 512	512 × 512	512 × 512
Rotation time (s)	0.25	0.33–0.5	0.4–0.5	0.5
Pitch	0.55	0.7–1.3	0.6–1.3	1.0
Contrast protocol	Thoracic CT angiography (dual-energy CECT, VNC)	Thoracic CT angiography (NCCT, CECT)	Thoracic CT angiography (NCCT, CECT)	Routine chest CT (NCCT, CECT)
Contrast amount, injection rate	90–100 mL, 3–4 mL/s	70–90 mL, 4–5 mL/s	70–90 mL, 4–5 mL/s	70–90 mL, 3 mL/s
Scan delay	10 s after bolus tracking	10 s after bolus tracking	10 s after bolus tracking	Fixed 60 s
**Reconstruction parameters**				
Slice thickness (mm)/increment (mm)	2/2	3/3	3/3	3/3
Kernel	Br40	B30f., Br40, Bv40	B	B
Iterative reconstruction	ADMIRE 3	ADMIRE 3	iDose 4	iDose 4

*Note* Siemens is located in Erlangen, Germany. Philips is located in Andover, Massachusetts. CECT = contrast-enhanced CT, VNC = virtual non-contrast CT, NCCT = non-contrast CT.

*Data are median with interquartile range in parentheses.

### Technical evaluation

**Figure 2:**
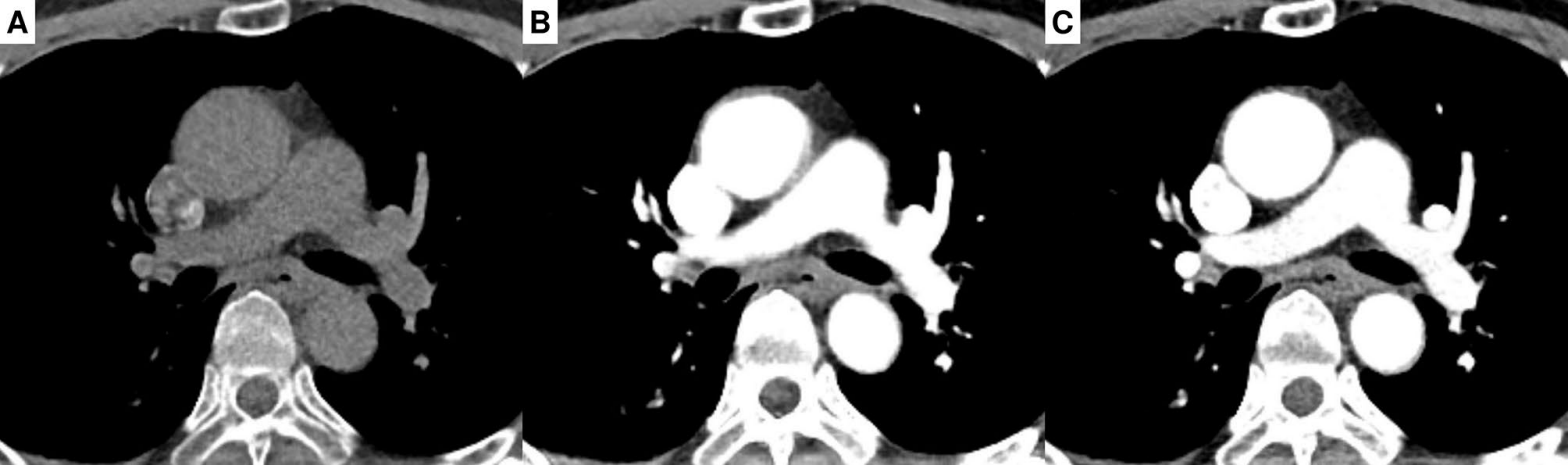
Images of a 63-year-old woman with right pneumothorax from the tuning set are presented. Non-contrast CT (**A**), synthetic contrast-enhanced CT (**B**), and contrast-enhanced CT (**C**).

**Figure 3:**
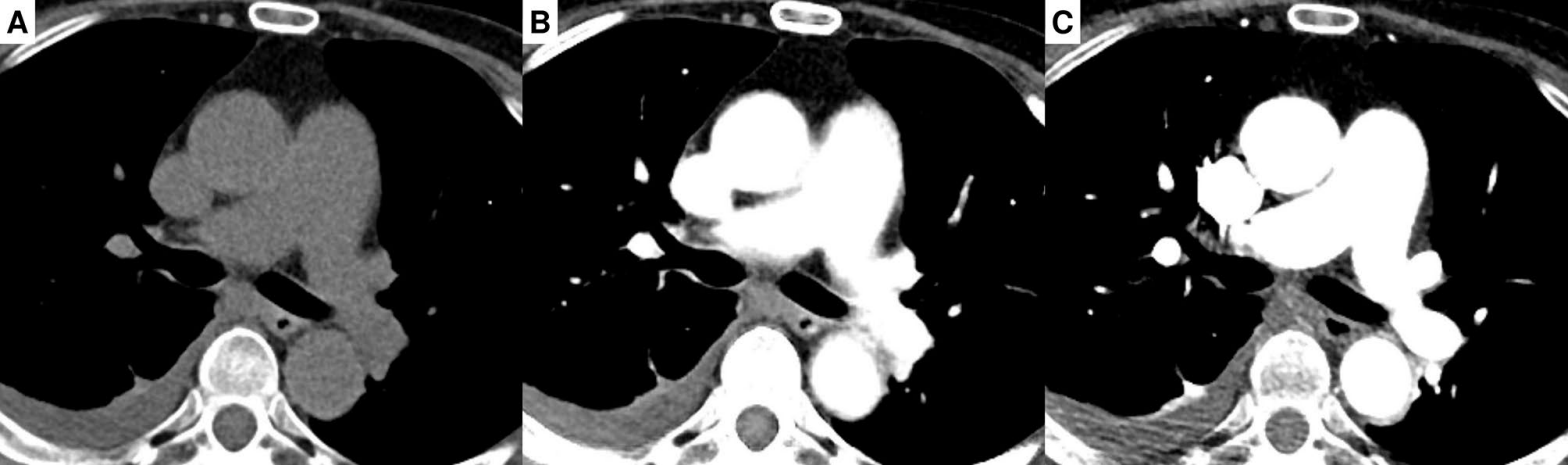
Images of a 65-year-old woman with right pleural effusion from test set 1 are presented. Non-contrast CT (**A**), synthetic contrast-enhanced CT (**B**), and contrast-enhanced CT (**C**).

**Figure 4:**
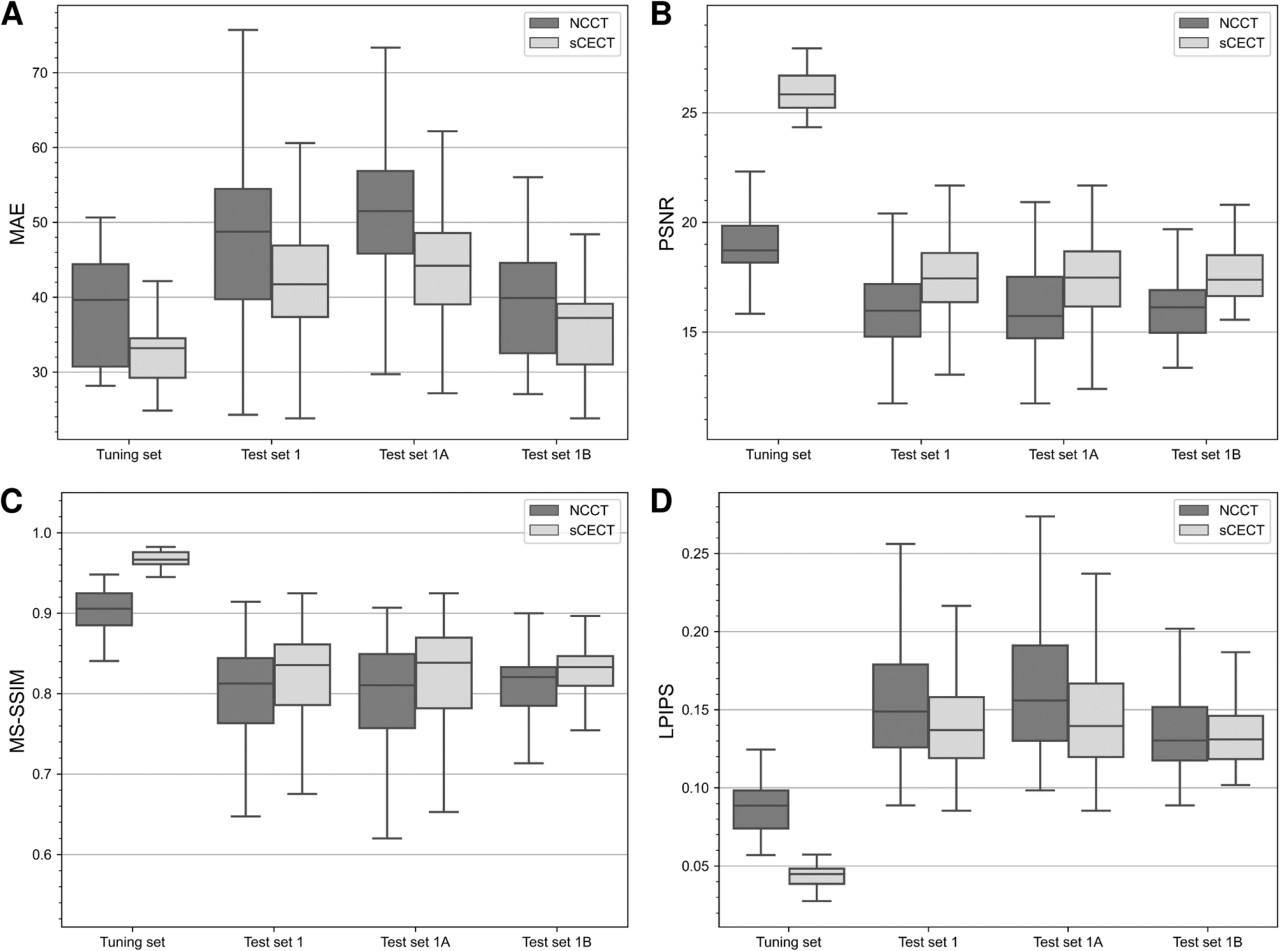
Comparison of image similarity metrics between non-contrast CT (NCCT) and synthetic contrast-enhanced CT (sCECT) with contrast-enhanced CT as the ground truth. Mean absolute error (MAE) (**A**), peak signal-to-noise ratio (PSNR) (**B**), multiscale structural similarity index measurement (MS-SSIM) (**C**), and learned perceptual image patch similarity metric (LPIPS) (**D**). Lower MAE, higher PSNR, higher MS-SSIM, and lower LPIPS values indicate higher image similarity. All comparisons showed significant differences (*P* < 0.05).

Examples of representative cases from the tuning set and test set 1 are shown in Figs. 2 and 3, respectively. The sCECT images showed significantly higher similarity to the ground-truth CECT than NCCT in all quantitative metrics in both the tuning set and test set 1 (Fig. 4, Table 2). In the tuning set, the sCECT images showed a lower median MAE (33.19 [IQR, 29.24–34.53] vs 34.64 [IQR, 30.73–44.67]; *P* < 0.001), a higher median PSNR (25.84 [IQR, 25.22–26.70] vs 18.72 [IQR, 18.16–19.85]; *P* < 0.001), higher median MS-SSIM (0.97 [IQR, 0.96–0.98] vs 0.91 [IQR, 0.88–0.92]; *P* < 0.001), and lower median LPIPS (0.04 [IQR, 0.04–0.05] vs 0.09 [IQR, 0.07–0.10]; *P* < 0.001) than NCCT images. In test set 1, sCECT had a lower median MAE (41.72 [IQR, 37.36–46.90] vs 48.74 [IQR, 39.73–54.48]; *P* < 0.001), higher median PSNR (17.44 [IQR, 16.37–18.60] vs 15.97 [IQR, 14.79–17.19]; *P* < 0.001), higher median MS-SSIM (0.84 [IQR, 0.79–0.86] vs 0.81 [IQR, 0.76–0.84]; *P* < 0.001), and lower median LPIPS (0.14 [IQR, 0.12–0.16] vs 0.15 [IQR, 0.13–0.18]; *P* < 0.001) than NCCT. The findings were also similar in subsets of test set 1.

**Table 2:** Evaluation of quantitative similarity metrics.

Data set	CT	MAE ↓	PSNR ↑	MS-SSIM (× 100) ↑	LPIPS (× 100) ↓
Tuning set	VNC	34.64 (30.73–44.67)	18.72 (18.16–19.85)	90.56 (88.49–92.47)	8.87 (7.40–9.83)
	sCECT	33.19 (29.24–34.53)	25.84 (25.22–26.70)	96.66 (96.09–97.59)	4.49 (3.85–4.84)
		*P* < .001	*P* < .001	*P* < .001	*P* < .001
Test set 1	NCCT	48.74 (39.73–54.48)	15.97 (14.79–17.19)	81.26 (76.33–84.42)	14.89 (12.59–17.89)
	sCECT	41.72 (37.36–46.90)	17.44 (16.37–18.60)	83.55 (78.58–86.12)	13.70 (11.91–15.81)
		*P* < .001	*P* < .001	*P* < .001	*P* < .001
Test set 1A	NCCT	51.52 (45.83–56.88)	15.73 (14.72–17.51)	81.04 (75.71–84.91)	15.59 (13.01–19.13)
	sCECT	44.20 (39.04–48.60)	17.48 (16.16–18.68)	83.85 (78.19–86.96)	13.96 (11.97–16.68)
		*P* < .001	*P* < .001	*P* < .001	*P* < .001
Test set 1B	NCCT	39.89 (32.52–44.61)	16.12 (14.96–16.91)	82.05 (78.49–83.30)	13.03 (11.75–15.17)
	sCECT	37.22 (30.98–39.12)	17.39 (16.64–18.50)	83.30 (80.97–84.67)	13.10 (11.85–14.61)
		*P* < .001	*P* < .001	*P* < .001	*P* = .003

*Note* Data are median (interquartile range). Lower MAE, higher PSNR, higher MS-SSIM, and lower LPIPS values indicate higher image similarity. VNC = virtual non-contrast CT, sCECT = synthetic contrast-enhanced CT, NCCT = non-contrast CT, MAE = mean absolute error, PSNR = peak signal-to-noise ratio, MS-SSIM = multiscale structural similarity index measurement, LPIPS = learned perceptual image patch similarity metric.

### Performance in depicting mediastinal lymph nodes

**Figure 5:**
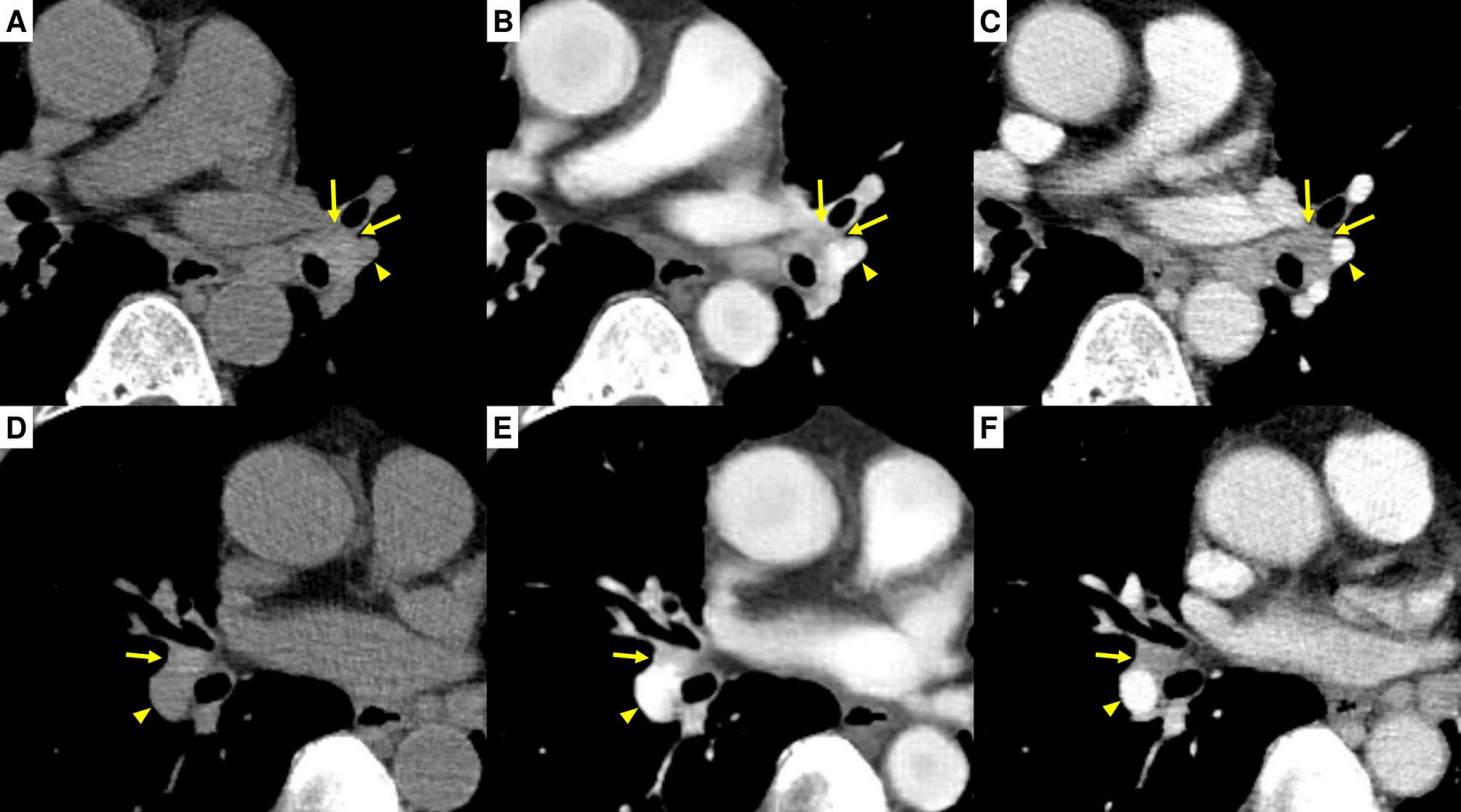
Images of a 78-year-old man with lung cancer and multiple mediastinal lymph node metastases from test set 2. Non-contrast CT (**A**, **D**), synthetic contrast-enhanced CT (**B**, **E**), and contrast-enhanced CT (**C**, **F**). Hilar lymph nodes (arrows), which are clearly visible on contrast-enhanced CT, are better distinguished from adjacent pulmonary vessels (arrowheads) on synthetic contrast-enhanced CT than on non-contrast CT.

An example of a representative case from test set 2 is shown in Fig. 5. The median of the lesion CNR of the mediastinal lymph nodes and background noise in each node measurement (n = 55) in test set 2 calculated on CECT images were 4.60 (IQR, 3.79–5.79) and 18.95 (IQR, 16.66–20.92), respectively. The median lesion CNR in the sCECT group was higher than that in the NCCT group (5.00 [IQR, 1.97–10.25] vs 0.52 [IQR, 0.14–1.03]; *P* < 0.001), while the median background noise in the sCECT group was also higher than that in the NCCT group (18.88 [IQR, 17.38–21.56] vs 17.79 [IQR, 16.04–17.79]; *P* < 0.001). We did not statistically compare measurements between sCECT and CECT images because of the difference in degrees of contrast enhancement between the development set and test set 2 due to the CT protocols.

**Figure 6:**
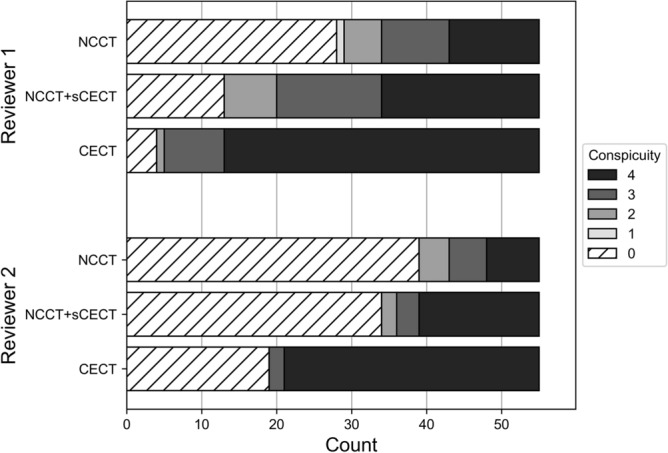
Comparison of radiologists’ ratings regarding lesion conspicuity of mediastinal lymph nodes in test set 2 between non-contrast CT (NCCT), non-contrast CT with synthetic contrast-enhanced CT (NCCT + sCECT), and contrast-enhanced CT (CECT). Undetected lesions are labeled as 0 and indicated by hatched bars.

In the observer study on test set 2, both reviewers detected a higher number of lymph nodes on NCCT with sCECT than on NCCT alone (reviewer 1, 76% [42 of 55 nodes] vs 49% [27 of 55 nodes], *P* = 0.003; reviewer 2, 38% [21 of 55 nodes] vs 29% [16 of 55 nodes], *P* = 0.06). The reader-averaged JAFROC FOMs calculated from NCCT alone, NCCT with sCECT, and CECT were 0.48, 0.52, and 0.68, respectively. There was no significant difference in JAFROC FOMs between the modalities (*P* = 0.059). The FROC curves from the three modalities are shown in Supplementary Fig. S2 Both reviewers had a higher lesion conspicuity rating for NCCT with sCECT compared to NCCT alone (*P* ≤ 0.001 for both), and both also rated CECT images higher in comparison to images of the other two groups (*P* < 0.001 for both; Fig. 6, Supplementary Table S1).

## Discussion

This study demonstrated the technical feasibility of deep learning-based synthetic contrast-enhanced CT (sCECT) in chest CT and evaluated the performance of this approach in depicting mediastinal lymph nodes. In patients with mediastinal lymphadenopathy, sCECT demonstrated a higher contrast-to-noise ratio of lymph nodes (6.15 vs 0.74; *P* < 0.001) than non-contrast CT (NCCT). In an observer study on the same patients, radiologists detected more lymph nodes (reviewer 1, 76% [42 of 55 nodes] vs 49% [27 of 55 nodes], *P* = 0.003; reviewer 2, 38% [21 of 55 nodes] vs 29% [16 of 55 nodes], *P* = 0.06) with higher lesion conspicuity (*P* ≤ 0.001) on NCCT with sCECT than on NCCT alone. The reader-averaged JAFROC FOMs calculated from NCCT alone, NCCT with sCECT, and CECT were 0.48, 0.52, and 0.68, respectively. There was no significant difference in JAFROC FOMs between the modalities (*P* = 0.059).

The most important strength of the current study is that we performed technical validation on a heterogeneous test set of CT data, including various CT vendors and scanning parameters. Many studies have shown deep learning applications of image-to-image synthesis in radiology, including cross-modality synthesis and reconstruction, but reports on external data are rare^3^. We believe that the quantitative performance of the proposed model shows the potential for generalizability, which is essential for any deep learning model to be used in clinical practice^22^.

Few previous studies have applied deep learning for synthetic contrast enhancement in CT. Santini et al.^5^ demonstrated synthetic enhancement in non-contrast cardiac CT to delineate the left cardiac chambers. Liu et al.^23^ proposed a deep learning model to generate synthetic enhancement of major arteries in non-contrast abdominopelvic CT. However, to our knowledge, there are no previous studies that have performed end-to-end conversion of a whole volume of NCCT into sCECT images. We believe that acquiring VNC CT in the development set played a crucial role in the successful training of the proposed model. Misalignment between non-contrast and ground-truth contrast-enhanced images is an obstacle in the development of synthetic contrast enhancement^24,25^. The VNC reconstruction of dual-energy CT enabled perfect spatial registration between the input and ground truth.

The observer study performed by two radiologists showed that the mediastinal lymph nodes were more conspicuous on sCECT than on NCCT, which can be attributed to the higher CNR of the lymph nodes. However, only one radiologist showed a statistically significant increase in the detection rate on sCECT images compared to NCCT images. The trained model relatively poorly delineated hilar and segmental lymph nodes adjacent to pulmonary vessels that are often difficult to detect on NCCT. Further training on a more heterogeneous group of patients with mediastinal lymphadenopathy may improve the model’s performance. Nonetheless, the proposed model successfully generated sCECT images with higher CNR in terms of technical feasibility.

Importantly, we do not claim that our deep learning implementation or methods to generate sCECT can replace CECT. The ultimate goal of our study on sCECT is to yield additional information, including improved lesion conspicuity and detectability, from NCCT, but not to predict the degree or pattern of contrast enhancement of the lesions. Not only does a vast majority of chest CT not require the use of contrast media, but also sCECT has a potential benefit in patients under certain conditions. These include allergy to iodinated contrast media, frequent CT examinations, chronic kidney disease, and poor vascular access. Additionally, we believe that sCECT can be utilized as a type of post-processing technique. A future application of sCECT is its use in automated volumetric segmentation and analysis. A previous study used synthetic non-contrast CT to improve the generalizability of CT segmentation tasks^26^. Likewise, sCECT may enable segmentation tools based on CECT to be generalized to NCCT data.

Our study has several limitations. First, our study included a small number of patients. However, such a number is reasonable as generative models demand high computational loads, unlike classification models. Several previous studies on image synthesis in radiology were also based on small study populations^18,27^. Second, we could not strictly control CT protocols and indications because of the retrospective nature of our study. The ideal training and test sets might have been patients with similar diseases and similar CT protocols. However, dual-energy CT with routine contrast amount or CT angiography for suspected lung cancer is not commonly performed in clinical practice. Lastly, our proposed model may not be an optimal deep learning approach for sCECT. Comparison and combination with different approaches including CNN (e.g., U-Net^7^) and generative models based on unpaired data (e.g., CycleGAN^28^) are warranted.

In conclusion, we implemented a deep learning model for generating synthetic contrast enhancement from non-contrast chest CT. Synthetic contrast-enhanced CT demonstrated good quantitative performance in terms of image similarity metrics and improved depiction of mediastinal lymph nodes. Applying the proposed deep learning model in clinical practice requires further studies on a larger population with more heterogeneous diseases.

## Supplementary Information

Supplementary Information.
