# Supplementary material for: Generating synthetic contrast enhancement from non-contrast chest computed tomography using a generative adversarial network
Source: Sci Rep. 2021 Oct 14;11:20403. doi: 10.1038/s41598-021-00058-3 (PMC8516920; doi:10.1038/s41598-021-00058-3)
Supplement: Supplementary file 1 — Supplementary Information. [file 41598_2021_58_MOESM1_ESM.pdf]

Supplementary Information for

Generating Synthetic Contrast Enhancement from Non-contrast Chest Computed Tomography Using a Generative Adversarial Network

Jae Won Choi, MD[1,2], Yeon Jin Cho, MD, PhD[1,2], Ji Young Ha, MD, PhD[3], Seul Bi Lee, MD[1], Seunghyun Lee, MD[1,2], Young Hun Choi, MD, PhD[1,2], Jung-Eun Cheon, MD, PhD[1,2,4], Woo Sun Kim, MD, PhD[1,2,4]

[1]Department of Radiology, Seoul National University Hospital, 101 Daehak-ro, Jongno-gu, Seoul, 03080, Korea

[2]Department of Radiology, Seoul National University College of Medicine, 103 Daehak-ro, Jongno-gu, Seoul, 03080, Korea

[3]Department of Radiology, Gyeongsang National University Changwon Hospital, Changwon, 51472, Korea

[4]Institute of Radiation Medicine, Seoul National University Medical Research Center, 103 Daehak-ro, Jongno-gu, Seoul, 03080, Korea

Figure S1

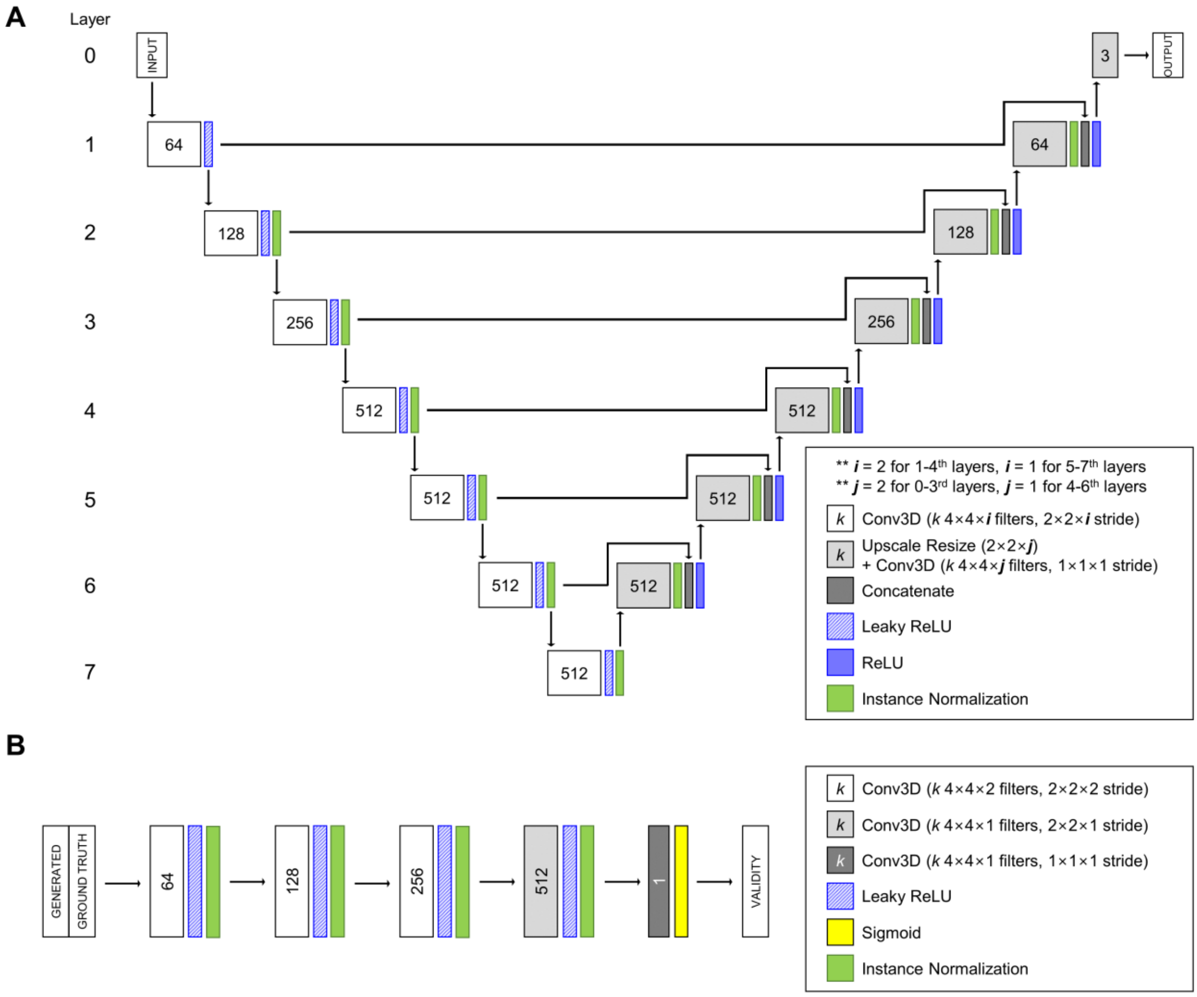

Our deep learning model is a 3D implementation of the pix2pix model that consists of a U-Net generator network (A) and a PatchGAN discriminator network (B).

Figure S2

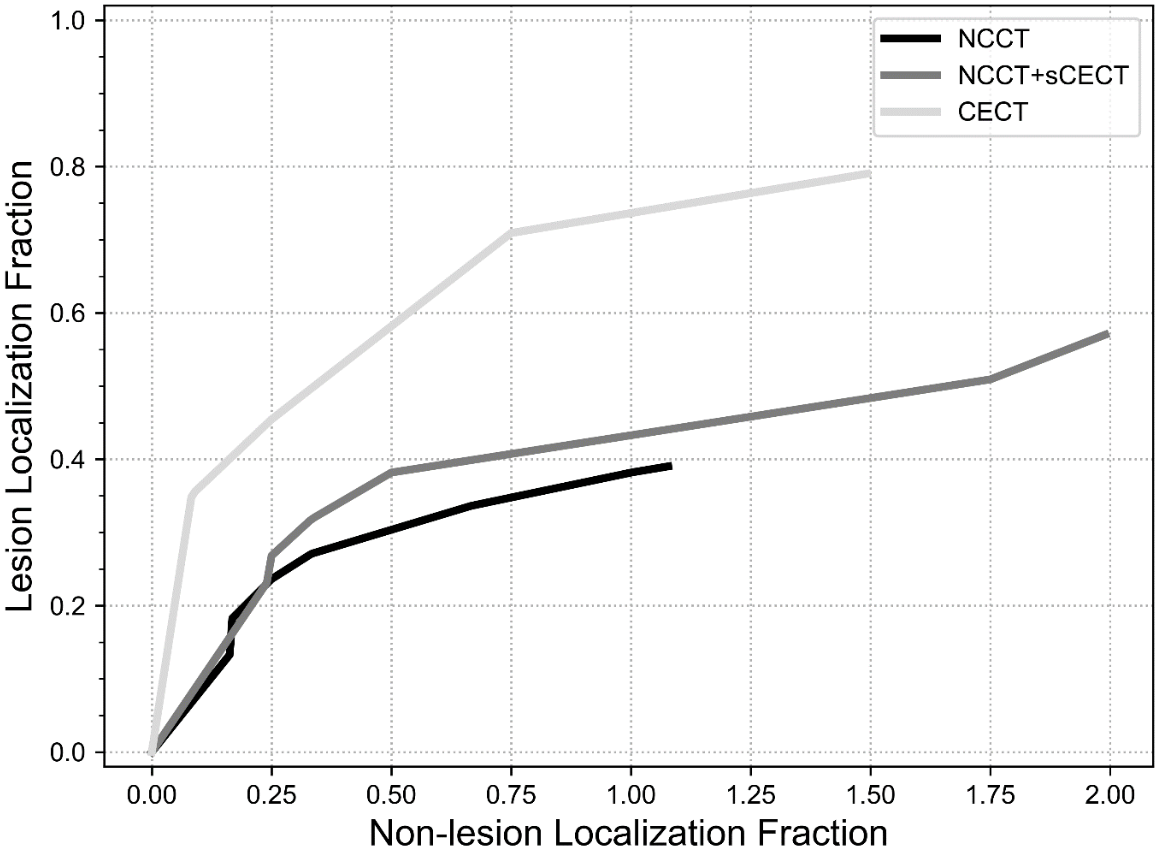

Reader-averaged free-response receiver operating characteristic (FROC) curves of lymph node detection in non-contrast CT (NCCT), non-contrast CT with synthetic contrast-enhanced CT (NCCT+sCECT), and contrast-enhanced CT (CECT).

Table S1. Conspicuity of Mediastinal Lymph Nodes in the Observer Study

	Lesion conspicuity	Number of lymph nodes			*P*-value		
		1: NCCT	2: NCCT + sCECT	3: CECT	1 vs 2	2 vs 3	1 vs 3
Reviewer 1							
	> 0	49 (27/55)	76 (42/55)	93 (51/55)	.001*	< .001*	< .001*
	1	2 (1/55)	0 (0/55)	0 (0/55)			
	2	9 (5/55)	13 (7/55)	2 (1/55)			
	3	16 (9/55)	26 (14/55)	15 (8/55)			
	4	22 (12/55)	38 (21/55)	76 (42/55)			
	0	51 (28/55)	24 (13/55)	7 (4/55)	.003†	.04†	< .001†
Reviewer 2							
	> 0	29 (16/55)	38.2 (21/55)	65.5 (36/55)	< .001*	< .001*	< .001*
	1	0 (0/55)	0 (0/55)	0 (0/55)			
	2	7 (4/55)	4 (2/55)	0 (0/55)			
	3	9 (5/55)	6 (3/55)	4 (2/55)			
	4	13 (7/55)	29 (16/55)	62 (34/55)			
	0	71 (39/55)	38 (34/55)	35 (19/55)	.06†	.002†	< .001†

Note.—Data are percentages with number of lymph nodes in parentheses. Undetected lesions are labeled as 0.
NCCT = non-contrast CT, sCECT = synthetic contrast-enhanced CT, CECT = contrast-enhanced CT.
* Differences in the lesion conspicuity are compared using the Wilcoxon signed-rank test.
† Detection rates are compared using the McNemar test.
